# Work-related distress among interpreters working in interpreter-mediated psychotherapy: a 1-year follow-up

**DOI:** 10.3389/fpsyg.2026.1883184

**Published:** 2026-10-14

**Authors:** Monja Lucia Herold, Rita Rosner

**Affiliations:** Department of Psychology, Catholic University of Eichstätt-Ingolstadt, Eichstätt, Germany

**Keywords:** interpreter-mediated psychotherapy, longitudinal study, perceived work-related distress, posttraumatic stress symptoms, trauma-informed interpreting competence

## Abstract

**Background:**

Interpreter-mediated psychotherapy may pose demands on interpreters, particularly in trauma-focused settings. Empirical evidence on interpreters’ perceived work-related distress and associated variables remains limited. This longitudinal exploratory study examined perceived work-related distress and posttraumatic stress symptoms (PTSS) among interpreters participating in a trauma-focused cognitive behavioral therapy (TF-CBT) workshop and explored associations with psychotherapy-related difficulties, trauma-informed interpreting competence, and involvement in trauma-focused psychotherapy.

**Method:**

The analytical sample comprised 68 interpreters working in psychotherapy, who contributed up to three observations each, yielding a total of 120 valid observations of perceived work-related distress. Observed outcome data were available for 51 participants at baseline (T0), 37 at 6 months (T2), and 32 at 12 months (T3). Perceived work-related distress was assessed using a single author-developed item, PTSS using the PCL-5, and process- and content-related interpreting difficulties and trauma-informed interpreting competence using author-developed self-report items. Involvement in trauma-focused psychotherapy was also assessed. Linear mixed-effects models were used.

**Results:**

Perceived work-related distress was moderate and did not significantly change over time. Higher PTSS (*B* = 0.22, SE = 0.10, *p* = 0.034) and greater process-related difficulties (*B* = 0.34, SE = 0.10, *p* < 0.001) were associated with higher perceived work-related distress. Content-related difficulties, trauma-informed interpreting competence, and involvement in trauma-focused psychotherapy were not significant (all *p* ≥ 0.137).

**Discussion:**

The findings suggest that process-related difficulties may be particularly relevant to interpreter well-being. Role agreements between therapists and interpreters, structured communication, collaboration within the therapeutic triad, and training or supervision addressing process-related challenges may represent relevant areas for practice and future research.

## Introduction

Effective communication is a prerequisite for psychotherapeutic treatment. Approximately 14.1 million foreign nationals were registered in Germany’s Central Register of Foreigners at the end of 2024 (Statistisches Bundesamt, 2025). In addition, data from the Federal Office for Migration and Refugees (BAMF Bundesamt für Migration und Flüchtlinge, 2025) indicate that 106,298 initial asylum applications were received in Germany from January to November 2025. Accordingly, language barriers are relevant in psychotherapeutic care in Germany, with psychotherapists in training reporting that a proportion of their patients have limited German-language proficiency (Bachmann et al., 2026). In such cases, psychotherapeutic treatment often requires the involvement of an interpreter (Schreiter et al., 2016). When psychotherapist and patient do not share sufficient proficiency in a common language, an interpreter may be involved, transforming the traditional therapist–patient dyad into a therapeutic triad. Particularly in refugee care, access to appropriately qualified professional interpreters may be limited for some required languages or dialects, and non-professional or *ad hoc* interpreters may therefore also be involved in psychotherapeutic and psychosocial care (Geiling et al., 2021). Previous research suggests that psychotherapy involving interpreters can be effective, particularly in the treatment of trauma-affected refugee populations. Some studies found comparable treatment outcomes for psychotherapy conducted with and without interpreters (Ardenne et al., 2007; Brune et al., 2011), and a review of the limited available evidence similarly reported no consistent differences in treatment outcomes associated with interpreter use (Jensen et al., 2017). However, findings are not uniform, with one larger retrospective study reporting less improvement on several mental health outcomes among trauma-affected refugees receiving interpreter-mediated psychotherapy compared with those treated without an interpreter (Sander et al., 2019). Moreover, interpreter qualification may be relevant: professional and *ad hoc* interpreters should not be considered interchangeable, as untrained interpreters may be more likely to make errors with potential clinical consequences (Bauer and Alegría, 2010). Thus, the available evidence suggests that interpreter-mediated psychotherapy can be feasible and effective, while outcomes may depend on contextual factors, including the qualifications of interpreters and the quality of collaboration within the therapeutic triad. However, it still comes with challenges for all involved. The quality of collaboration between therapist, interpreter, and patient is a central determinant of the success of interpreter-mediated psychotherapy. This therapeutic triad differs from the traditional therapist–patient dyad and introduces specific relational and communicative demands, including the clarification of roles and responsibilities, coordination of communication within the triad, management of potential misunderstandings, and consideration of culturally specific aspects of communication (Morina et al., 2010). A stable working alliance within the triad has been identified as crucial for therapeutic processes and outcomes, whereas relational imbalances, role conflicts, and insufficient collaboration between the therapist and the interpreter may disrupt treatment (Gryesten et al., 2023; Hanft-Robert and Mösko, 2024).

These triadic dynamics place substantial demands on interpreters, who are required to navigate complex role expectations, maintain neutrality while engaging empathically, and manage culturally sensitive and emotionally distressing content. Studies report that unclear role definitions, asymmetric alliances (e.g., stronger patient–interpreter bonds), and difficulties in collaboration can lead to insecurity, emotional distress, and increased cognitive and affective distress for interpreters (Gryesten et al., 2023; Metzner et al., 2018; Miller et al., 2005). Although interpreters are often expected to function as neutral linguistic conduits, empirical evidence indicates that they routinely assume additional roles, such as cultural mediation or relational facilitation, which may enhance therapeutic communication but can also intensify responsibility and perceived distress if expectations remain ambiguous (Hanft-Robert et al., 2018; Sleptsova et al., 2014).

Overall, the literature suggests that interpreters are not passive participants but active contributors to the therapeutic process. Research on the perceived work-related distress of interpreters in psychotherapy has mainly been qualitative (Fennig and Denov, 2021; Geiling et al., 2021). These studies indicate that involvement in psychotherapy, particularly trauma-focused therapy, can be a significant stressor.

Across the helping professions, evidence suggests that pre-existing post-traumatic stress symptoms (PTSS) or a personal trauma history increase vulnerability to secondary traumatic stress (STS). Whereas PTSS refer to an individual’s own trauma-related symptoms resulting from previous traumatic experiences, STS describes trauma-related stress reactions that arise from indirect exposure to the traumatic experiences of others (American Psychiatric Association, 2022; Figley, 1995). Meta-analytic and systematic reviews among mental health professionals report small but consistent associations between prior trauma exposure or PTSS and elevated STS or vicarious traumatization (Henderson et al., 2025; Hensel et al., 2015).

The limited evidence regarding interpreters points in a similar direction. Approximately 10–15% of interpreters report clinically relevant PTSS (Kindermann et al., 2017; Mehus and Becher, 2016; Teegen and Gönnenwein, 2002), a prevalence slightly higher than that reported for the general population in Germany (Hauffa et al., 2011). In psychotherapy-related interpreting, greater PTSS symptom severity and prior trauma exposure have been associated with higher levels of secondary traumatic stress (Kindermann et al., 2017), a pattern also observed in multiprofessional refugee-care samples including interpreters (Denkinger et al., 2018). Reviews and cross-sectional studies further identify personal trauma history or forced displacement as relevant vulnerability factors for secondary traumatization among interpreters, although findings vary by context and operationalization (Geiling et al., 2021; Hapfelmeier et al., 2025).

Based on previous evidence linking PTSS to adverse psychological outcomes, PTSS were examined as a potential associated variable of perceived work-related distress. Overall, systematic quantitative research examining the association between PTSS and perceived work-related distress among interpreters remains scarce.

In Germany, interpreting in healthcare and mental healthcare is not subject to uniform nationwide qualification requirements. Consequently, interpreters working in these settings may differ considerably in their professional qualifications, ranging from academically trained or state-certified interpreters to individuals who have completed shorter community-interpreting programs or have no formal interpreting qualification (Bundesverband der Dolmetscher und Übersetzer, 2023). Professional standards emphasize that interpreting in healthcare requires specific interpreting competencies and relevant contextual knowledge (Bundesverband der Dolmetscher und Übersetzer, 2023). For psychosocial and psychotherapeutic settings, guidelines additionally emphasize specific qualification for working in the therapeutic triad and define minimum standards for qualified language mediation in counseling and therapy (Bundesweite Arbeitsgemeinschaft der Psychosozialen Zentren für Flüchtlinge und Folteropfer (BAfF), 2022). Empirical studies consistently show that lay and *ad hoc* interpreters make significantly more clinically relevant interpreting errors than professionally trained interpreters (Bauer and Alegría, 2010; Flores et al., 2012). Insufficient training and lack of professional standards are associated with interpreting errors, omissions, inappropriate comments, and misunderstandings of psychiatric terminology, which may lead to misdiagnosis and inadequate treatment (Bauer and Alegría, 2010; Fennig and Denov, 2021; Hanft-Robert and Mösko, 2024). These issues are especially prevalent with *ad hoc* interpreters and pose challenges for the interpreter as well as for the therapist (Bauer and Alegría, 2010; Chen and Chen, 2020; Hunt and Swartz, 2017; Morina et al., 2010). Beyond linguistic challenges, interpreters frequently report role ambiguity and uncertainty in triadic therapeutic settings, which may result in deviations from neutral interpretation, especially when working with trauma-related content as is noted in qualitative studies (Metzner et al., 2018; Morina et al., 2010). Moreover, insufficient collaboration within the therapeutic triad can hinder communication and the therapeutic process, whereas clear role definitions and effective collaboration facilitate treatment (Gryesten et al., 2023; Hanft-Robert and Mösko, 2024; Miller et al., 2005).

Trauma-informed interpreting competence may be associated with lower perceived work-related distress. Effective interpreting in psychotherapy requires specific competencies, including knowledge about mental disorders, professional role adherence, confidentiality, and emotional distance (Metzner et al., 2018; Morina et al., 2010). Interpreters often translate the entire therapeutic process, which may expose them to emotionally distressing content (Gartley and Due, 2017; Geiling et al., 2021; Hassan and Blackwood, 2021). In this context, interpreters benefit from understanding psychological symptoms such as PTSS and the principles of trauma-focused therapy (Abdallah-Steinkopff, 1999; Morina et al., 2010). Lack of knowledge about therapy methods or conceptualizations of psychotherapy that do not align with those of the therapist have been described as a source of stress (Morina et al., 2010). Similarly, a clear understanding of agreement on the roles of all involved is crucial and can give the interpreter security (Becher and Wieling, 2015; Hassan and Blackwood, 2021). Additionally, behaviors regarding confidentiality and neutrality are of high importance for interpreters (Morina et al., 2010). However, little is known about whether trauma-informed interpreting competence is associated with perceived work-related distress among interpreters working in psychotherapy.

Involvement in trauma-focused psychotherapy has consistently been described as a factor associated with perceived work-related distress associated with interpreting (Hassan and Blackwood, 2021; Mehus and Becher, 2016). Geiling et al. (2022) found that a high percentage of traumatic content was related to higher perceived work-related distress and work-related exhaustion in interpreters. There is research on the influence of high trauma load on STS (Hapfelmeier et al., 2025; Kindermann et al., 2017), but little is known about whether involvement in trauma-focused psychotherapy is associated with perceived work-related distress among interpreters.

Despite this extensive qualitative literature, there is little longitudinal quantitative evidence on whether perceived linguistic difficulties, role ambiguity, and challenges related to trauma-focused interpreting are associated with perceived work-related distress among interpreters.

The present longitudinal exploratory study therefore aimed to investigate perceived work-related distress and associated variables among interpreters currently working in psychotherapy who participated in a TF-CBT training workshop over a 12-month period. Perceived work-related distress was assessed at baseline, 6-month follow-up, and 12-month follow-up. Furthermore, we examined whether PTSS, perceived process-related interpreting difficulties, trauma-informed interpreting competence, and involvement in trauma-focused psychotherapy were associated with perceived work-related distress over time. Accordingly, the following research question was addressed: (1) Are PTSS, perceived interpreting difficulties, trauma-informed interpreting competence, and involvement in trauma-focused psychotherapy concurrently associated with perceived work-related distress over time? Given the exploratory nature of the study, the following research questions were addressed:

1. How does perceived work-related distress among interpreters working in psychotherapy develop over the 12-month study period?
2. Are PTSS, process- and content-related interpreting difficulties, trauma-informed interpreting competence, and involvement in trauma-focused psychotherapy associated with perceived work-related distress over time?

## Method

### Study design

This longitudinal exploratory study investigated perceived work-related distress among interpreters working in psychotherapy who participated in a TF-CBT training workshop. The study was conducted as part of Project BETTER CARE (Rosner et al., 2020). Data were collected at four measurement occasions: baseline before the training workshop (T0), immediately after the workshop (T1), 6 months after the workshop (T2), and 12 months after the workshop (T3). The present longitudinal analyses included T0, T2, and T3. The training was delivered in nine separate workshops conducted between September 2020 and June 2021. Each workshop lasted approximately 4 h and was attended by 10–15 interpreters; each participant attended one workshop. The research protocol was approved by the Institutional Review Board of the Catholic University Eichstätt-Ingolstadt on November 6, 2019 (approval no. 005–19). Written informed consent was obtained from all participants prior to study participation.

### TF-CBT interpreter workshop

The interpreters were trained to work in Trauma-Focused Cognitive Behavioral Therapy (TF-CBT; Cohen et al., 2017) as part of Project BETTER CARE (Rosner et al., 2020) and consisted of a single four-hour session. The workshops were facilitated by the first author together with colleagues involved in the BETTER CARE project, as described in Müller et al. (2023). Due to COVID-19-related restrictions, the workshop was delivered online. Participation was open to individuals working as community interpreters who were interested in interpreting in trauma-focused cognitive behavioral therapy (TF-CBT); formal interpreter training or certification was not required for participation. The workshop was aimed to prepare interpreters for their role in TF-CBT. Training covered trauma-related background (e.g., traumatic experiences, flight, stress, PTSD symptoms, and diagnostics), collaboration with therapists, and the core components of trauma-focused cognitive behavioral therapy (TF-CBT) according to Cohen et al. (2009). These included psychoeducation about trauma and trauma reactions, parenting skills, relaxation and affect-regulation strategies, cognitive coping, development and processing of a trauma narrative, *in vivo* mastery of trauma reminders, conjoint child–parent sessions, and enhancing safety and future development. The workshop also included practical exercises and discussions related to the interpreters’ role in TF-CBT (Müller et al., 2023).

### Participants and recruitment

Participants were recruited via social organizations and Facebook groups. Participation in the workshop was open to individuals working as community interpreters who were interested in interpreting in TF-CBT; formal interpreter training or certification was not required. A total of 129 interpreters participated in the BETTER CARE workshop. At baseline, participants were asked whether they were currently interpreting in psychotherapy. Sixty-eight participants reported interpreting in psychotherapeutic settings at the time of the baseline assessment and therefore completed the psychotherapy-specific questionnaires. The present longitudinal analytical sample therefore comprised these 68 participants, that were interpreting in psychotherapy. A detailed overview of recruitment, eligibility, exclusions, and follow-up participation is presented in Figure 1.

**Figure 1:**
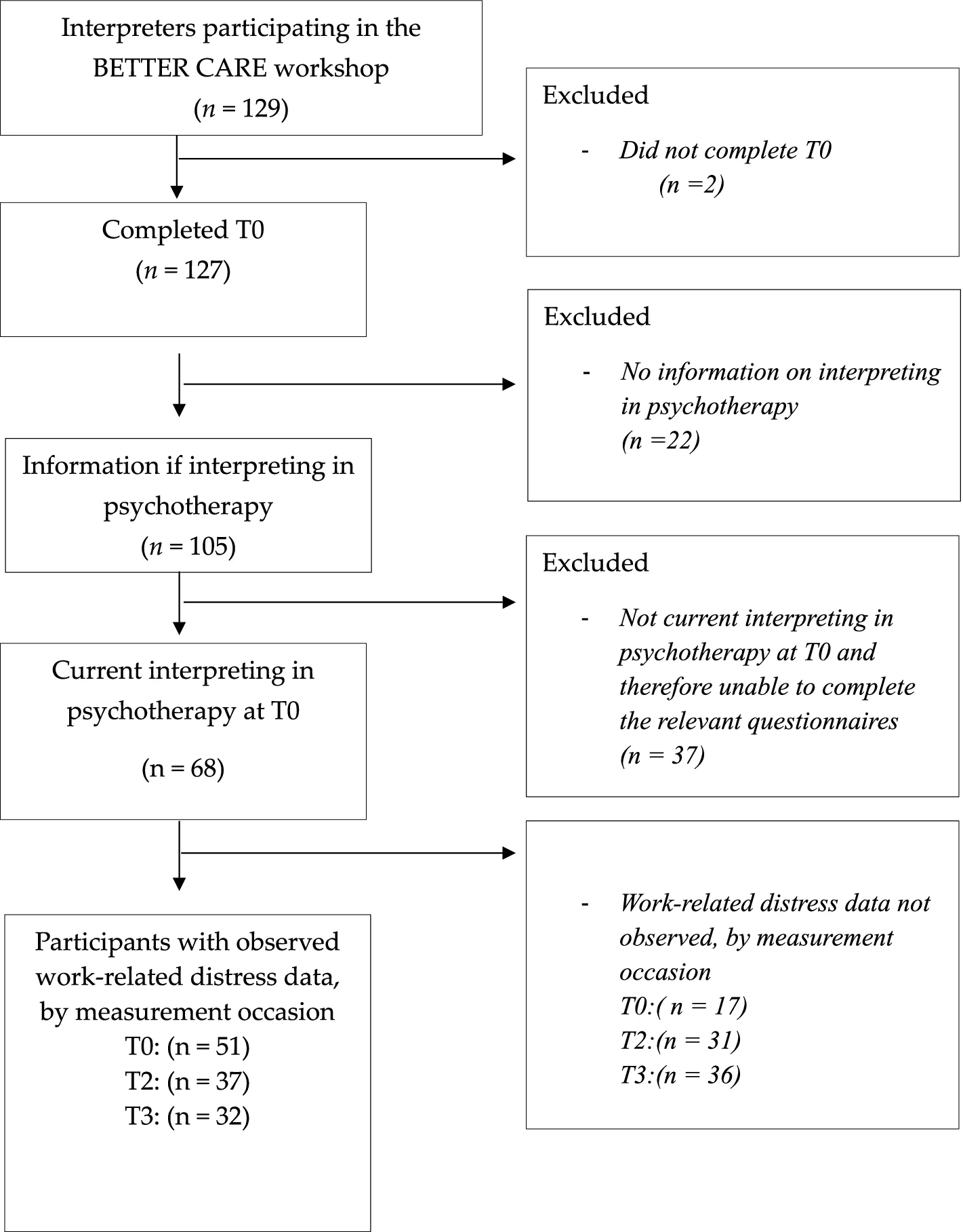
Diagram of participants flow.

### Instruments

#### Demographic information

Sociodemographic and work-related information was assessed using author-developed self-report items covering age, gender, country of origin, religion, occupation, educational level, professional experience as an interpreter, own refugee experience, primary field of work, and the language(s) interpreted.

#### Involvement in trauma-focused psychotherapy

Participants were asked using a dichotomous item whether they were currently interpreting in trauma-focused psychotherapy (yes/no). Involvement in trauma-focused psychotherapy was assessed at T0, T2, and T3. For the purpose of the present study, trauma-focused psychotherapy was described to interpreters in accessible, non-technical language to ensure a shared understanding of the concept. The following definition was provided: “Some individuals experience extremely distressing events in their lives, such as violence, the sudden death of friends or family members, or sexual abuse. These experiences are referred to as traumatic experiences. Some people who have experienced a traumatic event subsequently develop posttraumatic stress disorder (PTSD). Individuals with PTSD may repeatedly experience distressing images or nightmares related to the traumatic event, withdraw socially, feel anxious or irritable, and avoid reminders of the trauma. Trauma-focused therapy is a form of psychotherapy in which these traumatic experiences are explicitly addressed and processed with the aim of alleviating symptoms and improving patients’ wellbeing”.

#### Perceived work-related distress

In the present study, perceived work-related distress is conceptualized as a broad measure of distress associated with interpreting in psychotherapy rather than as a measure or indicator of secondary traumatic stress. Perceived work-related distress was assessed using a single self-report item developed for the present study. Participants were asked: “How burdened do you feel by your work as an interpreter in psychotherapy?” Responses were recorded on a 5-point Likert scale ranging from 1 (not at all) to 5 (very much), with higher scores indicating greater perceived work-related distress. The item assessed participants’ current perceived burden related to their work as an interpreter in psychotherapy and was administered at baseline (T0), 6-month follow-up (T2), and 12-month follow-up (T3).

#### Posttraumatic stress symptoms (PTSS)

Posttraumatic stress symptoms (PTSS) were assessed using the PTSD Checklist for DSM-5 (PCL-5; Weathers et al., 2013; Krüger-Gottschalk et al., 2017), a 20-item self-report questionnaire based on the DSM-5 criteria for PTSD (American Psychiatric Association, 2013). Participants completed the PCL-5 with reference to the event they had identified as the most distressing in the Life Events Checklist (LEC). They were instructed to rate their symptoms during the past month. Items assess symptoms such as repeated, disturbing memories of the stressful experience and avoidance of thoughts or feelings related to the experience. Items are rated on a 5-point Likert scale ranging from 0 (“not at all”) to 4 (“extremely”). A total PTSS score was calculated by summing the scores across all 20 items, resulting in a possible total score ranging from 0 to 80, with higher scores indicating greater posttraumatic stress symptom severity. The authors suggest a total cut-off score for a probable PTSD diagnosis between 31 and 33. For the present study, a cut-off score of ≥ 33 was applied to identify participants with clinically relevant posttraumatic stress symptom levels. Internal consistency was high in the present sample (McDonald’s ω = 0.96). The PCL-5 was administered at T0, T2, and T3.

#### Difficulties in the interpreting process

Based on the existing literature (e.g., Morina et al., 2010; Tribe and Morrissey, 2004; Miller et al., 2005), commonly reported difficulties experienced by interpreters in trauma-focused psychotherapy were operationalized as exploratory self-report items. The items captured, on the one hand, linguistic challenges and satisfaction with one’s own interpreting performance and, on the other hand, role and task clarity as well as the handling of trauma-related content during interpretation. All items were rated on a five-point Likert-type scale ranging from 1 (“does not apply at all”) to 5 (“fully applies”). The individual indicators were combined into a composite measure because they were conceptualized as complementary manifestations of process-related challenges encountered during interpreter-mediated psychotherapy. Specifically, linguistic difficulties, uncertainty regarding role expectations, and perceived quality of collaboration all reflect different aspects of the interpreter’s experience within the therapeutic process rather than independent constructs. This conceptual overlap was supported empirically by the substantial correlation between language-related and role-related difficulties (r = 0.66 at baseline). Furthermore, given the exploratory nature of the study and the relatively small sample size, a parsimonious modelling approach was considered preferable to estimating multiple highly related predictors simultaneously. Accordingly, this composite should be regarded as an exploratory index reflecting process-related interpreting difficulties rather than as a fully validated psychometric construct.

##### Process related difficulties

The items “Linguistic difficulties occurred during trauma-focused psychotherapy,” “I was satisfied with the quality of my interpretations during trauma-focused psychotherapy” (reverse-coded), and “My interpretations facilitated the therapeutic process during trauma-focused psychotherapy” (reverse-coded) focused on the interpreting process and difficulties in the interpreting task. The items “There were moments in trauma-focused psychotherapy in which I was unsure what was expected of me” and “Coordination between the patient, the therapist, and myself worked well during trauma-focused psychotherapy” (reverse-coded) should assess difficulties in role clarity. These items were therefore combined into an exploratory composite index of process-related interpreting difficulties. The internal consistency of this composite was acceptable (Cronbach’s α = 0.69). Because the items were expected to contribute unequally to the construct, McDonald’s omega was additionally calculated and indicated good reliability (ω = 0.84). Although these reliability indices indicate acceptable internal consistency, they do not establish construct validity. As the composite has not yet undergone comprehensive psychometric validation (e.g., factor analytic evaluation or assessments of convergent and discriminant validity), findings based on this index should be interpreted with appropriate caution.

##### Content-related difficulties

The item “During trauma-focused psychotherapy, I translated some words differently in order to avoid placing additional distress on the patient” was included to assess avoidance of interpreting trauma-related content.

The items focusing on difficulties in the interpreting process were administered at T0, T2 and T3. The wording of these items was adapted to participants’ current interpreting context. Participants involved in trauma-focused psychotherapy answered the items with reference to trauma-focused psychotherapy, whereas participants not involved in trauma-focused psychotherapy answered the corresponding items with reference to psychotherapy more generally. Although the specific therapeutic content could differ between trauma-focused and other forms of psychotherapy, all items referred to the same overarching setting of interpreter-mediated psychotherapy. The process-related items addressed aspects such as linguistic difficulties, interpreting performance, role clarity, and collaboration within the therapeutic triad, which were considered applicable across both contexts. The same response format and scoring procedure were therefore used for both groups.

#### Trauma-informed interpreting competence

##### Competence

Items assessing trauma-informed interpreting competence were developed by the authors to assess interpreters’ role clarity, helpful attitudes toward psychotherapy, and knowledge about PTSD. Item development was informed by an extensive literature review (Gartley and Due, 2017; Hunt and Swartz, 2017; Miller et al., 2005; Morina et al., 2010; Tribe and Morrissey, 2004). The resulting items were intended as exploratory indicators of foundational trauma-informed interpreting competence. Eight items assessed role clarity, confidentiality obligations, and other professional behaviors considered essential for interpreters working in trauma-focused psychotherapy. Example items included: “Some content is culturally inappropriate, so I did not translate it”; “It is important for psychotherapy to clarify who takes on which roles in the conversation”; “The patient should not be reminded too strongly of the traumatic experience during the therapeutic conversation” (reverse-coded); and “I worry that the therapist might think I am a bad interpreter if I translate unclear patient statements word for word” (reverse-coded). The items were rated on a five-point Likert-type scale ranging from 1 (“does not apply at all”) to 5 (“fully apply”). The internal consistency of the role competence items was moderate (Cronbach’s α = 0.63). Because the items were expected to contribute unequally to the construct, McDonald’s omega was additionally calculated and yielded a comparable estimate (ω = 0.67). Although these reliability indices indicate acceptable internal consistency, they do not provide evidence of construct validity.

##### Knowledge

Knowledge of PTSD was assessed using a shortened and interpreter-adapted version of the 40-item TF-CBT-Web knowledge test, originally developed to monitor participants’ knowledge in the web-based training program TF-CBT-Web (Heck et al., 2015). Four single-choice items captured knowledge about PTSD. For example, participants were asked, “Which of the following is NOT a typical symptom of posttraumatic stress disorder (PTSD)?”, with response options including avoidance of trauma-related situations, re-experiencing (e.g., distressing memories or nightmares), negative changes in thoughts and mood, and loss of appetite. For each item, participants selected one response option, which was scored as correct or incorrect.

Both the role competence items and the PTSD knowledge items were administered at T0, T1, T2 and T3: prior to the workshop, immediately after the workshop, and at 6-month and 12-month follow-up. The two components were considered complementary dimensions of foundational trauma-informed interpreting competence, capturing professional role-related attitudes as well as PTSD knowledge regarded as prerequisites for competent interpreting in trauma-focused psychotherapy. Standardized scores were therefore combined into an exploratory composite index of trauma-informed interpreting competence. The composite competence score was subsequently z-standardized using the mean and standard deviation of all available observations across measurement occasions in the analytic sample. Thus, standardization was based on the pooled distribution across measurement occasions rather than being performed separately within each measurement occasion. Because the composite assessed foundational knowledge and attitudes rather than applied behavioral skills, it should be interpreted as an exploratory composite index rather than a validated psychometric scale. Furthermore, this composite has not yet undergone comprehensive psychometric validation (e.g., factor analytic evaluation or assessments of convergent and discriminant validity). Consequently, findings based on this index should be interpreted with appropriate caution and require confirmation in future studies using validated measures.

### Data collection

Questionnaires were administered online using Qualtrics (2005). T0 questionnaires were completed within 2 weeks before the workshop and T1 questionnaires within 2 weeks after the workshop. Follow-up assessments were conducted 6 months (T2) and 12 months (T3) after the workshop. Participants received €20 vouchers after completing each survey. Written informed consent was obtained prior to participation.

Given the short interval between T0 and T1 (maximum approximately 4 weeks), perceived work-related distress, which referred to participants’ interpreting work in psychotherapy in general, was not reassessed at T1 but was assessed again at the longer-term follow-ups (T2 and T3). Accordingly, the present longitudinal analyses were based on T0, T2, and T3.

### Data analysis

Of the 129 interpreters who participated in the BETTER CARE workshop, 68 reported at baseline that they were currently interpreting psychotherapy and were therefore eligible for the present study. The final analytical sample comprised these 68 interpreters. Because linear mixed-effects models accommodate unbalanced longitudinal data, participants contributed data whenever valid outcome information was available. Consequently, 51, 37, and 32 valid outcome observations were available at T0, T2, and T3, respectively (120 observations in total). To examine potential attrition effects, baseline perceived work-related distress and PTSS were compared between participants who provided an observed work-related distress outcome at least once during follow-up (T2 or T3) and those who provided no observed work-related distress outcome at either follow-up. Group differences were examined using Welch’s t-tests. Because linear mixed-effects models can accommodate unbalanced longitudinal data, participants were included if they contributed valid outcome data at one or more measurement occasions.

Statistical analyses were conducted in R (R Core Team, 2020) using RStudio (Posit Team, 2024). Linear mixed-effects models were estimated using the 120 available observations with valid outcome data. To ensure comparability across hierarchical model comparisons, the same analytical dataset was used for all models. Because linear mixed-effects models accommodate unbalanced longitudinal data, participants were not required to contribute data at every measurement occasion.

Descriptive statistics were calculated for all study variables at each measurement occasion to summarize distributions and examine temporal trends. Because descriptive statistics at each measurement occasion were based on all available observations, the participants contributing data differed across T0, T2, and T3. Consequently, differences in occasion-specific means should be interpreted descriptively and not as within-person trajectories. Pearson correlation coefficients were calculated for baseline data using pairwise complete observations to avoid dependency among repeated measurements within participants. Continuous predictor variables were *z*-standardized to facilitate interpretation and comparability of effect sizes.

Measurement time point was coded as a continuous predictor (1 = T0 [baseline], 2 = T2 [6 months after the workshop], and 3 = T3 [12 months after the workshop]). The post-training assessment (T1) was not included in the present analyses because the predictor variables examined in this study were not assessed at that measurement occasion. Accordingly, the regression coefficient for measurement time point represents the estimated average linear change in perceived work-related distress across the three analyzed measurement time points.

Hierarchical linear mixed-effects models were estimated using the R packages lme4 (Bates et al., 2015) and lmerTest (Kuznetsova et al., 2017). Repeated measurements (Level 1) were nested within participants (Level 2), and all models included a random intercept for participant, whereas all predictor variables were specified as fixed effects. Perceived work-related distress served as the dependent variable in all models. It was assessed at each measurement occasion using a single-item five-point Likert-type scale. Two parallel versions of the item were used, depending on participants’ current work context: “How burdened do you currently feel by your work as an interpreter in psychotherapy?” and “How burdened do you currently feel by your work as an interpreter in trauma-focused psychotherapy?” Participants received the version corresponding to their current work setting at each assessment. Thus, the two versions differed only in their contextual reference and were intended to assess the same underlying construct of work-related distress. Given the approximately continuous five-point response scale, the repeated-measures design, and the objective of modelling average changes in perceived work-related distress over time, linear mixed-effects models were considered appropriate. As a sensitivity analysis, an ordinal cumulative link mixed-effects model was additionally estimated using the R package ordinal with the same fixed- and random-effects structure to evaluate the robustness of the findings. Models were estimated using maximum likelihood (ML; REML = FALSE). Denominator degrees of freedom and *p* values were obtained using the Satterthwaite approximation implemented in the lmerTest package (Kuznetsova et al., 2017).

Model building followed a hierarchical, theory-driven approach. A null model including only a random intercept for participants was estimated first to quantify between-person variance in perceived work-related distress. Measurement time point was subsequently added to examine changes in distress over time, followed by posttraumatic stress symptoms. In the next step, perceived interpreting difficulties were included. Because perceived interpreting difficulties and role clarity were highly correlated, these variables were combined into the composite score *interpreting process difficulties*. Finally, trauma-informed interpreting competence and involvement in trauma-focused psychotherapy were added to evaluate whether they accounted for additional variance in perceived work-related distress. Random slopes were not estimated because the relatively small sample size and limited number of repeated observations did not support more complex random-effects structures.

Because all nested models were estimated on the same analytical dataset, model comparisons were performed using likelihood-ratio tests based on differences in −2 log-likelihood values.

Model summaries were inspected to evaluate fixed effects, random effects, and overall model fit. Multicollinearity was considered unproblematic because variance inflation factor (VIF) values were close to 1 (Akinwande et al., 2015). Model assumptions were evaluated using residual diagnostics assessing linearity, normality, homoscedasticity, autocorrelation, multicollinearity, and influential observations. No substantial violations of model assumptions were detected.

Because perceived work-related distress was assessed using a single Likert-type item with ordered response categories, we conducted an ordinal cumulative link mixed-effects model as a sensitivity analysis to examine whether the findings were robust to modelling the outcome as an ordinal rather than a continuous variable. The ordinal model used the same fixed- and random-effects structure as the primary linear mixed-effects model. The model yielded the same overall pattern of results. Higher posttraumatic stress symptoms (*B* = 0.42, *SE* = 0.19, 95% CI [0.05, 0.80], *p* = 0.026) and greater process-related difficulties (*B* = 0.74, *SE* = 0.21, 95% CI [0.33, 1.15], *p* < 0.001) were significantly associated with higher perceived work-related distress. Measurement time point (*p* = 0.076), content-related difficulties (*p* = 0.387), trauma-informed interpreting competence (*p* = 0.533), and previous involvement in trauma-focused psychotherapy (*p* = 0.353) were not significantly associated with perceived work-related distress. These findings support the robustness of the primary analyses (see Appendix A).

As an additional sensitivity analysis, multiple imputation was used to assess the robustness of the primary available-data analysis to missing questionnaire responses. Across 68 participants and three scheduled measurement occasions, 204 person-occasion observations were theoretically possible. Of these, 22 person-occasion records were entirely absent from the original longitudinal dataset (T0: *n* = 3; T2: *n* = 5; T3: *n* = 14). Entirely missed assessment occasions (*n* = 22) were not included in the imputation procedure. These occasions reflected nonparticipation in the respective assessment wave and were distinguished from questionnaire nonresponse within an otherwise observed person-occasion record. We did not generate additional records for entirely missed assessment waves because the purpose of the sensitivity analysis was to evaluate the robustness of the primary results to missing questionnaire data within observed assessment occasions, rather than to impute outcomes for complete wave nonresponse. Following removal of one duplicate person-occasion record identified during data verification, 182 unique person-occasion records remained. Of these, 120 contained observed values for perceived work-related distress, process-related difficulties, and content-related difficulties, whereas these three variables were jointly missing in 62 records because the corresponding questionnaire had not been completed (T0: *n* = 14; T2: *n* = 26; T3: *n* = 22). Thus, the 120 observed outcomes in the primary analysis and the 182 records in the multiple-imputation sensitivity analysis refer to different stages of the same longitudinal data structure (see Appendix B1). Multiple imputation was performed using 20 imputed datasets and 50 iterations using the mice package (van Buuren and Groothuis-Oudshoorn, 2011). Two-level predictive mean matching (2L.pmm) was used for perceived work-related distress, process-related difficulties, and content-related difficulties. Participant ID was specified as the level-2 clustering variable in the imputation models, and measurement occasion was included as a level-1 predictor. PTSS, trauma-informed interpreting competence, and involvement in trauma-focused psychotherapy were complete within the 182 existing person-occasion records and were included as predictors but were not imputed. Predictive mean matching ensured that imputed values were drawn from observed values; consequently, imputed perceived work-related distress remained within the five permissible response categories (1–5). Entirely absent person-occasion records were not generated for imputation. The same linear mixed-effects model used in the primary analysis was fitted separately to each of the 20 imputed datasets, including a participant-level random intercept. Parameter estimates and standard errors were pooled across imputations using Rubin’s rules (Rubin, 1987).

The multiple-imputation analysis yielded estimates that were generally similar in direction and magnitude to those of the primary available-data analysis. Process-related difficulties remained significantly associated with greater perceived work-related distress (*B* = 0.35, SE = 0.11, 95% CI [0.14, 0.56], *p* = 0.001). The association with PTSS remained positive but was attenuated and no longer reached conventional statistical significance (*B* = 0.18, SE = 0.10, 95% CI [−0.02, 0.38], *p* = 0.075). Measurement occasion, content-related difficulties, trauma-informed interpreting competence, and involvement in trauma-focused psychotherapy remained non-significant. Thus, the sensitivity analysis supported the robustness of the association between process-related difficulties and perceived work-related distress, whereas the independent association with PTSS was less robust to the handling of missing data (see Appendix B2).

Sensitivity analyses were also conducted to examine whether the findings depended on combining conceptually distinct components into exploratory composite indices. When process-related difficulties were separated into interpreting-related and role-related difficulties, both associations with perceived work-related distress were positive, although neither reached statistical significance (interpreting-related difficulties: *B* = 0.23, SE = 0.12, 95% CI [−0.01, 0.48], *p* = 0.061; role-related difficulties: *B* = 0.18, SE = 0.12, 95% CI [−0.06, 0.43], *p* = 0.142). Thus, the statistically significant association observed for the composite index was not replicated for either component separately.

In a second sensitivity analysis, trauma-informed interpreting competence was separated into role-related attitudes/competence and PTSD knowledge. Neither component was significantly associated with perceived work-related distress (*B* = −0.02, SE = 0.16, *p* = 0.894; *B* = 0.14, SE = 0.14, *p* = 0.349, respectively). Due to missing component-level data, this analysis included 82 observations from 55 participants. Re-estimating the original model in this same subsample yielded a comparable pattern of results, suggesting that the findings were not substantially altered by separating the competence components.

### Description of the sample

Descriptive characteristics refer to participants who answered the respective baseline questionnaire and interpreted psychotherapy (*n* = 68). Owing to missing responses, the number of available observations varied across variables. Participants had a mean age of 46.63 years (SD = 14.40; range = 19–87; *n* = 52). Of those who reported gender (*n* = 65), 64.6% (*n* = 42) were female and 35.4% (*n* = 23) were male. Participants had lived in Germany for a mean of 18.17 years (SD = 12.45; range = 0–56; *n* = 64).

Regarding education level (*n* = 65), 10.8% (*n* = 7) reported no professional qualification, 35.4% (*n* = 23) had completed an apprenticeship or vocational training, 26.2% (*n* = 17) held a bachelor’s degree, 20.0% (*n* = 13) held a master’s degree, 6.2% (*n* = 4) reported another qualification, and 1.5% (*n* = 1) held a doctoral degree (PhD). Participants reported a mean professional experience of 5.99 years (SD = 5.41; range = 0–30; *n* = 62). Information on interpreter-specific qualification or training was available for 65 participants. Of these, 25 (38.5%) reported having an interpreter qualification or having received interpreter-specific training, whereas 40 (61.5%) reported no such qualification or training.

With respect to psychotherapy experience (*n* = 65), 35.4% (*n* = 23) reported prior personal experience with psychotherapy, 56.9% (*n* = 37) reported no prior psychotherapy experience, and 7.7% (*n* = 5) preferred not to disclose this information. Of the sample, 63.1% (*n* = 41) reported that they had interpreted at least one trauma-focused psychotherapy session, whereas 36.9% (*n* = 24) had not. Among participants who reported the number of interpreted psychotherapy sessions (*n* = 37), the number ranged from 1 to 250 sessions (*M* = 32.16, SD = 53.19).

## Results

Although the analytical sample comprised 68 participants, the number of participants providing observed perceived work-related distress data varied across measurement occasions. Valid outcome data were available for 51 participants at T0, 37 at T2, and 32 at T3. Detailed information on missingness and the data structure is provided in Appendix Table B1.

### Perceived work-related distress

*N* = 68 participants were assessed, of whom 51 provided a valid response to the perceived work-related distress item at baseline. Among these 51 participants, 27.45% (*n* = 14) reported very low levels of distress, 31.37% (*n* = 16) reported low levels, 15.69% (*n* = 8) reported moderate levels, 21.57% (*n* = 11) reported elevated distress, and 3.92% (*n* = 2) reported very high distress (Table 1).

**Table 1:** Participants’ sociodemographic characteristics at T0.

Parameters	*n*	*M* (SD)	*n* (%)
Age in years	52	46.63 (14.40)	
Gender	65		
Male			23 (35.4)
Female			42 (64.6)
Professional experience, years	62	5.99 (5.41)	
Own Refugee experience	65		19 (29.2)
Length of stay in Germany, years	64	18.17 (12.45)	
Number of traumatic life events	65	7.78 (6.46)	
Any interpreter qualification or training	65		25 (38.5)
Involvement in trauma-focused psychotherapy	65		41 (63.1)
Number of interpreted trauma-focused psychotherapy sessions	37	32.16 (53.19)	

Descriptive statistics refer to the baseline assessment. The number of observations (n) varies across variables because of missing responses. Descriptive statistics refer to the baseline assessment. The number of observations (n) varies across variables because of missing responses. Values are presented as M (SD) for continuous variables and n (%) for categorical variables. Participants’ sociodemographic characteristics at T0.

### Posttraumatic stress symptoms (PTSS)

With regard to posttraumatic stress symptoms (PTSS), 35.38% of participants (*n* = 23) scored at or above the applied cut-off of 33, indicating clinically relevant symptom levels.

### Interpreting difficulties

Descriptive statistics for perceived interpreting difficulties, trauma-informed interpreting competence, and previous involvement in trauma-focused psychotherapy are presented in Tables 2, 3. Overall, interpreters reported relatively low levels of process-related interpreting difficulties and moderate levels of content-related difficulties.

**Table 2:** Descriptive statistics (M, SD) for perceived work-related distress, PTSS, process-related difficulties, and the components of trauma-informed interpreting competence across time.

MTP	T0		T2		T3	
Variable	*n*	*M (SD)*	*n*	*M (SD)*	*n*	*M (SD)*
Perceived work-related distress	51	2.43 (1.2)	37	2.11 (1.02)	32	2.12 (1.21)
PTSS	65	27.8 (14.9)	64	30.3 (18.7)	54	31.8 (15.9)
Difficulties	51	2.20 (0.73)	37	1.99 (0.7)	32	2.0 (0.64)
Trauma-informed competence						
Competence	65	24.2 (5.81)	64	33.3 (4.41)	54	32.9 (5.9)
Knowledge	65	2.15 (1.25)	64	2.16 (1.42)	54	2.17 (1.28)

M = mean; SD = standard deviation. T0, T2, and T3 represent measurement time points 1, 2, and 3, respectively. Perceived work-related distress reflects overall perceived work-related distress. PTSS = posttraumatic stress symptoms. Difficulties refers to overall difficulties score. Higher scores indicate higher levels of the respective constructs. Trauma-informed interpreting competence used in the mixed-effects models was operationalized as an exploratory composite index derived from standardized role competence and PTSD knowledge scores. The two components are presented separately here for descriptive purposes only.

**Table 3:** Means, standard deviations, and zero-order correlations among the study variables at baseline.

Variable	*M*	*SD*	1	2	3	4	5	6
1. Distress	2.43	1.22						
2. PTSS	27.77	14.95	0.25					
			[−0.03, 0.49]					
3. Difficulties role clarity	2.16	0.91	0.24	0.24				
			[−0.04, 0.48]	[−0.04, 0.48]				
4. Difficulties language	2.16	0.82	0.19	0.20	0.66**			
			[−0.09, 0.45]	[−0.08, 0.45]	[0.46, 0.79]			
5. Difficulties content	2.39	1.46	0.17	0.13	0.11	0.15		
			[−0.11, 0.43]	[−0.15, 0.39]	[−0.17, 0.38]	[−0.13, 0.41]		
6. Competence	−0.08	1.01	0.08	0.13	−0.09	−0.18	0.32*	
			[−0.20, 0.35]	[−0.12, 0.36]	[−0.36, 0.19]	[−0.44, 0.10]	[0.04, 0.54]	
7. Trauma-focused therapy	0.63	0.49	0.03	−0.12	−0.17	−0.23	0.08	−0.11
			[−0.25, 0.30]	[−0.35, 0.13]	[−0.43, 0.11]	[−0.47, 0.05]	[−0.20, 0.34]	[−0.34, 0.14]

M and SD are used to represent mean and standard deviation, respectively. Values in square brackets indicate the 95% confidence interval for each correlation. The confidence interval is a plausible range of population correlations that could have caused the sample correlation (Cumming, 2014). Correlations were calculated using pairwise complete observations; pairwise sample sizes ranged from *n* = 51 to *n* = 65, * indicates *p* < 0.05. ** indicates *p* < 0.01. Distress: perceived work-related distress, Difficulties content: content-related difficulties, Competence: trauma-informed interpreter competence.

### Trauma-informed interpreting competence

Role competence and PTSD knowledge also indicated moderate levels at baseline. As shown in Table 2, most descriptive values varied only modestly across measurement occasions. Trauma-informed interpreting competence showed descriptively higher values at follow-up than at baseline. These descriptive differences may reflect competence gains associated with participation in the workshop, as previously examined by Müller et al. (2023). However, the descriptive findings of the present analysis alone do not allow for causal conclusions regarding the effect of the workshop. These descriptive differences should be interpreted cautiously because different participants contributed data at each assessment and the means therefore do not represent complete-case within-person trajectories.

### Involvement in trauma-focused psychotherapy

Regarding prior experience with trauma-focused psychotherapy, the majority of participants had already worked as interpreters in this specific therapeutic context at baseline. At T0, 41 participants (63.08%) reported having previously interpreted in trauma-focused psychotherapy, whereas 24 participants (36.92%) reported no such experience. Thus, while all participants had experience interpreting in psychotherapeutic settings, approximately two-thirds had specific prior experience with trauma-focused psychotherapy.

### Correlations between study variables

Table 3 presents baseline means, standard deviations, and zero-order correlations for the variables included in the correlational analyses. For trauma-informed interpreting competence, the table reports the standardized composite score that was used in the correlational and mixed-effects analyses, whereas the descriptive raw component scores (role competence and PTSD knowledge) are presented separately in Table 2. The strongest association was observed between role clarity difficulties and language-related difficulties (*r* = 0.66, *p* < 0.01), indicating substantial conceptual overlap between these two dimensions. Based on their theoretical overlap, their substantial empirical association (*r* = 0.66), and the aim of avoiding overparameterisation in the mixed-effects models, both variables were combined into the exploratory composite index of process-related interpreting difficulties. In addition, trauma-informed interpreting competence was associated with difficulties avoidance (*r* = 0.32, *p* < 0.05). No other statistically significant correlations were observed.

### Mixed-effects model comparisons

Linear mixed-effects models with random intercepts for participants were estimated to account for the nested data structure, including 120 observations from 68 participants. The null model revealed substantial between-person variance in perceived work-related distress (σ^2^ = 0.27), supporting the use of a multilevel approach. The estimated overall mean level of perceived work-related distress was *M* = 2.25 (SE = 0.12). Adding measurement time point did not significantly improve model fit compared to the null model, χ^2^(1) = 2.21, *p* = 0.14, and the fixed effect of measurement time point was not significant (*B* = −0.18, *SE* = 0.12, 95% CI [−0.41, 0.05], *p* = 0.14), indicating no systematic change in perceived work-related distress over time.

Including posttraumatic stress symptoms (PTSS; z-standardized) significantly improved model fit, χ^2^(1) = 6.69, *p* = 0.010. PTSS was significantly associated with higher perceived work-related distress (*B* = 0.29, *SE* = 0.11, 95% CI [0.08, 0.51], *p* = 0.009). When PTSS was included, the effect of measurement time point reached significance (*B* = −0.24, *SE* = 0.12, 95% CI [−0.48, −0.01], *p* = 0.048).

The addition of process-related and content-related difficulties further improved model fit, *χ^2^*(2) = 13.37, *p* = 0.001. Process-related difficulties were significantly and positively associated with perceived work-related distress (*B* = 0.34, *SE* = 0.10, 95% CI [0.14, 0.53], *p* < 0.001), whereas content-related difficulties were not significantly associated with perceived work-related distress (*B* = 0.13, *SE* = 0.10, 95% CI [−0.08, 0.33], *p* = 0.221). PTSS remained significantly associated with perceived work-related distress (*B* = 0.22, *SE* = 0.11, 95% CI [0.02, 0.43], *p* = 0.034), whereas measurement time point was not significantly associated with perceived work-related distress (*B* = −0.18, *SE* = 0.12, 95% CI [−0.42, 0.06], *p* = 0.137).

Trauma-informed interpreting competence was not significantly associated with perceived work-related distress (*B* = 0.08, *SE* = 0.11, 95% CI [−0.13, 0.29], *p* = 0.439). Similarly, involvement in trauma-focused psychotherapy was not significantly associated with perceived work-related distress (*B* = 0.32, *SE* = 0.35, 95% CI [−0.39, 1.03], *p* = 0.372).

Overall, the results indicate that higher post-traumatic stress symptoms and greater process-related difficulties show robust associations with perceived work-related distress, whereas measurement time points, content-related difficulties, trauma-informed interpreting competence, and trauma-focused psychotherapy did not show significant associations with perceived work-related distress (see Tables 4, 5). Participants with and without follow-up outcome data did not differ significantly in baseline perceived work-related distress (*M* = 2.56, SD = 1.24 vs. *M* = 2.21, SD = 1.18), Welch’s t(39.50) = −1.01, *p* = 0.319. Baseline PTSS also did not differ significantly between the groups, t(29.01) = −1.19, *p* = 0.245.

**Table 4:** Model comparison of linear mixed-effects models predicting perceived work-related distress.

Model	Predictors included	npar	AIC	BIC	logLik	deviance	DeltaAIC	Chisq	Df	*p*
Model 1	Intercept-only model	3	378.87	387.23	−186.44	372.8721	14.27			
Model 2	Model 1 + Measurement occasion	4	378.67	389.82	−185.33	370.6666	14.06	2.21	1	0.138
Model 3	Model 2 + Posttraumatic stress symptoms	5	373.98	387.91	−181.99	363.9752	9.37	6.69	1	0.010
Model 4	Model 3 + Process- and content-related difficulties	7	364.60	384.11	−175.30	350.6018	0.00	13.37	2	0.001
Model 5	Model 4 + Trauma-informed interpreting competence	8	365.92	388.22	−174.96	349.9152	1.31	0.69	1	0.407
Model 6	Model 5 + Involvement in trauma-focused psychotherapy	9	367.14	392.22	−174.57	349.1370	2.54	0.78	1	0.378

Models compared via likelihood-ratio tests; lower AIC indicates better fit. npar = number of estimated parameters; AIC = Akaike Information Criterion; BIC = Bayesian Information Criterion; logLik = log-likelihood; ΔAIC = difference in AIC relative to the best-fitting model; Chisq = likelihood ratio chi-square statistic; Df = degrees of freedom. Models were compared sequentially using likelihood ratio tests. Lower AIC and BIC values indicate better model fit.

**Table 5:** Hierarchical linear mixed-effects model predicting perceived work-related distress.

Predictor	*B*	SE	df	*t*	*p*	95% CI (low)	95% CI (high)
(Intercept)	2.552	0.239	110.77	10.67	< 0.001	2.078	3.026
MTP	−0.180	0.120	97.71	−1.50	0.137	−0.419	0.058
PTSS	0.224	0.105	105.33	2.15	0.034	0.017	0.432
Difficulties process	0.337	0.098	119.72	3.44	< 0.001	0.143	0.531
Difficulties content	0.125	0.101	116.41	1.23	0.221	−0.076	0.326
Competence	0.081	0.105	119.93	0.777	0.439	−0.126	0.290
Trauma-focused therapy	0.32	0.35	119.79	0.9	0.372	−0.393	1.027

B = unstandardized regression coefficient; SE = standard error; df = degrees of freedom; CI = 95% confidence interval. Perceived work-related distress was the dependent variable. MTP = measurement time point; PTSS = posttraumatic stress symptoms; Difficulties process: process-related difficulties, Difficulties content: content related difficulties, Competence: trauma-informed interpreter competence. Continuous predictors were z-standardized before analysis. Accordingly, coefficients refer to a one-standard-deviation increase in the respective predictor. The intercept and the binary predictor are reported on their original scales. Involvement in trauma-focused psychotherapy was coded as a binary variable (1 = no involvement, 2 = involvement).

## Discussion

To our knowledge the present study is the first to examine perceived work-related distress among interpreters working in psychotherapy longitudinally over multiple measurement points. Overall, the findings indicate predominantly low to moderate self-reported perceived work-related distress in the sample associated with interpreting in psychotherapy; however, the presence of elevated distress in a subset of interpreters highlights the need for cautious interpretation and underscores the relevance of monitoring psychological well-being even in apparently low-burdened groups. After 12 months, no significant changes in the level of perceived work-related distress were observed across the three measurement points. The strongest association was observed for difficulties regarding interpreting and role clarity, that is, the interpreting process. Given the exploratory nature of the study, these results should be interpreted cautiously; they suggest potential associations rather than causal relationships. Nonetheless, they provide important initial insights into factors that may be associated with interpreter well-being in psychotherapeutic settings.

The absence of a significant time effect may suggest that interpreters’ perceived work-related distress is shaped primarily by enduring job demands rather than short-term temporal fluctuations. However, this finding should be interpreted with caution, as the lack of a time effect may also reflect limited statistical power, measurement insensitivity, attrition, or the use of a single-item outcome measure. In addition, measurement occasions were scheduled relative to each participant’s workshop participation, with assessments conducted before and at defined intervals after the workshop. Thus, measurement time also reflected time since training, and potential training-related effects cannot be clearly separated from other changes over time. The absence of a significant time effect should therefore not be interpreted as evidence that the training had no effect on perceived work-related distress. The training may also have influenced the observed trajectories of the study variables. By providing interpreters with knowledge about trauma-focused psychotherapy and clarifying their professional role within the therapeutic setting, the workshop may have increased trauma-informed interpreting competence and reduced uncertainty regarding role expectations and the interpreting process. Such changes could, in turn, have contributed to lower perceived work-related distress or counteracted increases in distress associated with continued exposure to demanding psychotherapeutic work. Thus, the absence of a significant increase in work-related distress over time does not necessarily indicate that distress remained unaffected by the training. However, because all participants received the training and measurement occasions were defined relative to workshop participation, potential training-related changes cannot be distinguished from other changes occurring over time. These interpretations therefore remain tentative. Our interpretation nevertheless aligns with meta-analytic and longitudinal research across helping professions, which consistently links burnout and work-related distress to stable psychosocial work characteristics such as high demands, limited resources, and role ambiguity (Aronsson et al., 2017; Guthier et al., 2020; Lesener et al., 2020). Similar findings have been reported for psychotherapists, with longitudinal studies identifying stable burnout and well-being trajectories that are not primarily explained by situational stressors (van Hoy and Rzeszutek, 2023). Systematic reviews further emphasize the role of sociodemographic and work-related factors in therapist burnout and well-being (Lee et al., 2020; Simionato and Simpson, 2018). While empirical research on perceived work-related distress among interpreters in psychotherapeutic settings remains limited, the present study contributes longitudinal evidence on distress patterns in this specific professional context. Notably, data were collected during the COVID-19 pandemic; although the absence of a measurement time point effect during this period is consistent with the possibility that distress reflects relatively stable role- and task-related challenges, this interpretation should be considered tentative given the study’s methodological limitations.

In the primary available-data analysis, PTSS were significantly associated with perceived work-related distress after adjustment for the other predictors. However, this association was attenuated and did not reach conventional statistical significance in the multiple-imputation sensitivity analysis. Although the direction of the association was consistent across analyses, the evidence for an independent association between PTSS and perceived work-related distress should therefore be interpreted cautiously. Importantly, PTSS were assessed with reference to participants’ self-identified most distressing event and not specifically in relation to their interpreting work. Therefore, the observed PTSS cannot be attributed to trauma exposure in the interpreting context, and the association between PTSS and perceived work-related distress should not be interpreted as evidence that interpreting work contributed to the development of these symptoms. In contrast, the association between process-related difficulties and perceived work-related distress was robust across the primary and multiple-imputation analyses. Although the estimated association between PTSS and perceived work-related distress was attenuated after process-related and content-related difficulties were included in the hierarchical linear model, PTSS remained significantly associated with perceived work-related distress in the primary analysis. This pattern suggests that both PTSS and process-related challenges were independently associated with perceived work-related distress in psychotherapeutic interpreting. While previous research has reported associations between PTSS and secondary traumatic stress (Kindermann et al., 2017), the present findings also align with literature emphasizing the role of meaning-making and contextualization in therapeutic settings. Although initial exposure to traumatic material can be distressing, continued involvement in psychotherapy may facilitate normalization and cognitive integration of both clients’ and interpreters’ trauma-related experiences (Celik and Cheesman, 2018; Doherty et al., 2010; Miller et al., 2005; Splevins et al., 2010). Moreover, perceived work-related distress in this study was conceptualized broadly as distress associated with interpreting in psychotherapy rather than secondary traumatic stress per se, encompassing stressors unrelated to trauma exposure, such as translation-related challenges and ambiguities in role boundaries.

Prior research indicates that interpreters working in psychotherapeutic and mental health settings often lack formal training specifically tailored to clinical and emotionally demanding contexts, which may contribute to uncertainty in handling complex psychiatric terminology and role expectations. For example, lower rates of formal interpreting degrees have been reported among interpreters working in these settings (Geiling et al., 2022). Difficulties related to role boundaries were also reported at a moderate level, reflecting the structurally ambiguous position of interpreters within the therapeutic triad that has been widely described in the literature (Fennig and Denov, 2021; Hsieh, 2008; Searight and Searight, 2009). Difficulties related to interpreting traumatic content specifically showed greater variability, suggesting individual differences in how interpreters manage emotionally challenging material in therapeutic encounters.

Importantly, higher scores on the exploratory composite index of process-related interpreting difficulties were strongly associated with interpreters’ perceived work-related distress and showed the strongest association in the hierarchical linear model. This finding aligns with previous research demonstrating that mental health interpreting involves substantial cognitive, emotional, and interactional demands that have been associated with work-related distress (Metzner et al., 2018; Morina et al., 2010). By contrast, difficulties related to fears of harming the patient through addressing traumatic content were not associated with perceived work-related distress. As this item assessed strategic modification of translated content rather than distress resulting from trauma exposure per se, the absence of an association may indicate that such adaptations are not necessarily experienced as distressing. Instead, mitigation or reformulation of traumatic content may reflect deliberate professional strategies, either to protect the patient or to regulate the interpreter’s own emotional exposure. Previous studies suggest that interpreters engage in such practices as part of role negotiation within the therapeutic triad and as a means of managing emotional strain (Crezee et al., 2013; Geiling et al., 2021; Hsieh, 2010; Searight and Searight, 2009). In contrast, process-based challenges—such as managing role boundaries, interactional dynamics, and implicit expectations within psychotherapy— appear to be less controllable and were more strongly associated with interpreters’ perceived work-related distress in the present study. Insufficient training, lack of standardized qualifications, and limited access to supervision have been linked to interpreting errors, role confusion, and increased stress, potentially affecting both therapeutic processes and interpreters’ well-being (Bauer and Alegría, 2010; Denkinger et al., 2018; Hanft-Robert and Mösko, 2024). Overall, these findings suggest that interpreters’ distress in psychotherapeutic settings may be closely linked to context-specific process demands. However, because these demands were assessed using an exploratory composite index, this interpretation should be considered preliminary and requires replication using psychometrically validated measures.

In the present study, interpreter-related competencies were operationalized using an exploratory composite index integrating knowledge of PTSD, trauma-focused therapy, and trauma psychotherapy with competencies related to professional role clarity and collaboration within the therapeutic triad. The exploratory composite reflected foundational trauma-informed competence rather than the ability to apply such competencies in emotionally demanding psychotherapeutic situations and should therefore not be interpreted as a fully validated psychometric measure of trauma-informed competence. Accordingly, the absence of an association between this exploratory composite index and perceived work-related distress should not be interpreted as evidence that trauma-informed interpreting competence is irrelevant for interpreter well-being. Rather, given the exploratory nature of the study and the limited statistical power, this null finding should be regarded as preliminary and requires replication in larger, adequately powered samples. Instead, the findings may reflect the distinction between foundational competence and its application in clinical practice, as the composite assessed knowledge and professional role-related attitudes but not behavioral skills such as emotion regulation, communication strategies, or the flexible application of trauma-informed principles during psychotherapy sessions. Contemporary trauma-informed competency frameworks conceptualize competence as encompassing knowledge, skills, and attitudes (American Psychological Association, 2015). Accordingly, knowledge and role-related attitudes alone may not fully reflect professionals’ ability to apply trauma-informed principles in emotionally demanding clinical practice. This finding is consistent with the existing literature suggesting that psychoeducational and competence-based approaches tend to show small or limited effects on general perceived work-related distress, particularly when delivered as predominantly informational formats and in contexts characterized by high emotional and interactional demands (Donker et al., 2009). Accordingly, interpreter-related competencies may not be sufficient to directly buffer perceived work-related distress in psychotherapeutic settings. Rather, it is conceivable that such competencies primarily may be associated with more proximal process-related factors, such as role clarity, interactional security, or the ability to contextualize therapeutic material, which may in turn be associated with perceived work-related distress. Consistent with literature highlighting the role of collaboration and role clarity in interpreter-mediated psychotherapy (Fennig and Denov, 2021; Miller et al., 2005), future research should test whether interpreter-related competencies are linked to perceived work-related distress indirectly through process- and role-related mechanisms.

No statistically significant association was observed between involvement in trauma-focused psychotherapy and perceived work-related distress in the present study. Given the exploratory nature of the study and the relatively limited statistical power, this null finding should be interpreted cautiously and requires replication in larger, adequately powered longitudinal studies. This finding contrasts with evidence indicating a dose–response relationship between vicarious trauma exposure and STS of therapists, whereby higher trauma load—operationalized as greater work-related exposure or a higher percentage of working time with a trauma focus—is associated with increased STS (Cleary et al., 2024). At the same time, null findings have been reported in large clinician samples, showing that exposure to trauma narratives or the number of trauma-focused clients on caseload does not necessarily predict STS (Sodeke-Gregson et al., 2013).

Together, these mixed findings suggest that involvement in trauma-focused psychotherapy alone may be insufficient to capture clinically relevant trauma load. Variability in exposure intensity, cumulative burden, and moderating factors such as training and supervision may account for divergent results across studies (Hapfelmeier et al., 2025). Future research should therefore distinguish more clearly between categorical involvement and quantitative measures of trauma exposure when examining stress and secondary traumatization.

### Limitations

Several limitations should be considered when interpreting the findings of this study. First, the relatively small sample size may partly be related to the recruitment strategy and the specific target population addressed in this study. Recruitment focused on interpreters interested in facilitating psychotherapy for unaccompanied young refugees and was not conducted systematically across Germany. Thus, the recruitment channels used did not provide systematic coverage of the broader population of interpreters working in psychotherapeutic settings, and potentially eligible interpreters may not have been reached. In addition, recruitment through specific channels may have introduced self-selection, as interpreters with a particular interest in trauma-focused psychotherapy or further professional training may have been more likely to participate. These aspects of the recruitment strategy may have contributed to the relatively small sample size and should be considered when interpreting the findings and their generalizability. Accordingly, the findings should be interpreted as preliminary. In addition, participant self-selection may have introduced selection bias, as interpreters experiencing either particularly high or particularly low levels of perceived work-related distress may have been more likely to participate.

Second, all data were derived from self-report measures, which may be influenced by recall bias or social desirability. Moreover, relevant contextual and structural factors related to interpreters’ working conditions in psychotherapy (e.g., institutional context, workload, supervision, or employment status) were not systematically assessed, although these factors may be associated with psychological outcomes.

In addition, with the exception of the adapted PTSD knowledge items, the study relied primarily on newly developed exploratory questionnaires that had not yet undergone comprehensive psychometric validation. Perceived work-related distress was assessed using a single-item measure, which limits measurement precision, reliability, and construct coverage. Although single-item measures can reduce participant burden in longitudinal field studies, they cannot capture the multidimensional nature of perceived work-related distress. Therefore, the findings should be interpreted cautiously and should be replicated using validated multi-item instruments in future studies. The test–retest reliability of the single-item measure of perceived work-related distress was not assessed across the T0–T2 or T2–T3 intervals. Consequently, the temporal reliability and longitudinal comparability of this measure remain uncertain. This limitation should be considered when interpreting changes in perceived work-related distress over time. Above this, the single-item measure of work-related distress was administered in two context-specific versions, referring either to psychotherapy in general or specifically to trauma-focused psychotherapy. Although both versions were intended to assess the same overarching construct of perceived work-related distress, the different contextual reference may have influenced participants’ responses, as distress associated with trauma-focused psychotherapy may differ from distress experienced in psychotherapy more generally. Thus, some variability in work-related distress scores may reflect differences in the therapeutic context to which participants referred rather than differences in the underlying level of work-related distress. In addition, participants who changed their work setting during the study could receive a different item version at later assessments, potentially reducing longitudinal comparability. This measurement heterogeneity may have introduced additional variability into the analyses and should therefore be considered when interpreting the observed associations and changes over time.

The composite scores for competence and difficulties were constructed for exploratory purposes based on conceptually related items rather than established instruments and therefore require cautious interpretation. Although the process-related difficulties composite showed acceptable internal consistency (Cronbach’s α = 0.69; McDonald’s ω = 0.84), and the trauma-informed interpreting competence composite demonstrated acceptable reliability for an exploratory measure (Cronbach’s α = 0.63; McDonald’s ω = 0.67), internal consistency alone does not establish construct validity. Both composites were newly developed for the present study and have not yet undergone comprehensive psychometric validation, including factor analytic evaluation or assessments of convergent and discriminant validity. Although the composite index was significantly associated with perceived work-related distress in the primary and multiple-imputation analyses, neither interpreting-related nor role-related difficulties reached statistical significance when entered as separate predictors in the sensitivity analysis. Thus, the statistically significant finding depended on combining these components, and the individual contribution of each component remains uncertain. Furthermore, the absence of a significant association between the composite index of trauma-informed interpreting competence and perceived work-related distress should not be interpreted as evidence that interpreting competence is unrelated to distress. The index combined conceptually distinct aspects, including role-related attitudes and PTSD knowledge, and demonstrated relatively low internal consistency (Cronbach’s α = 0.63). These measurement limitations may have affected the ability to detect associations between specific competence domains and perceived work-related distress.

Consequently, they should be regarded as exploratory indices, and their construct validity remains to be established. A further limitation concerns the exploratory composite indices used in the present study. Some conceptually distinguishable aspects were combined to represent process-related difficulties and trauma-informed interpreting competence. Although this approach reduced model complexity in the context of the limited sample size, correlations between components do not establish unidimensionality. The composite indices should therefore be regarded as exploratory rather than as validated psychometric scales. Future studies with larger samples should examine their dimensional structure and psychometric properties and assess the individual components separately where appropriate. The composite assessed foundational knowledge and role-related attitudes and should therefore not be interpreted as a comprehensive measure of applied trauma-informed interpreting competence. At the time the present study was designed and the data were collected, no validated quantitative instruments assessing these constructs in interpreters working in psychotherapy were available. Therefore, theory-based items were developed based on the existing literature. Geiling et al. (2023) developed and evaluated the Role Conflicts Questionnaire for Interpreters (RoCo-I) to assess role conflicts experienced by interpreters working with refugees and in mental health care settings. The evaluation showed good psychometric properties, including satisfactory reliability and validity, and identified common conflicts between professional neutrality, emotional involvement, and advocacy for clients. Because the RoCo-I was published after the present study had been conducted, it could not be used in the current investigation. Future studies should employ validated instruments such as the RoCo-I where appropriate. Furthermore, although PTSS and perceived work-related distress represent conceptually distinct constructs, both were assessed using self-report measures and may partially overlap, which should be considered when interpreting their association.

Furthermore, the exploratory study design without a control group does not allow causal conclusions. Although data were collected longitudinally, the observational design does not permit conclusions about the temporal direction of the associations between PTSS, interpreting-related difficulties, and perceived work-related distress. Because time-varying predictors were analyzed concurrently with the outcome and within-person and between-person effects were not separated (e.g., by person-mean centering), the observed associations should not be interpreted as evidence that changes in the predictors lead to subsequent changes in work-related distress. We also did not assess whether interpreters received psychological or psychotherapeutic support during the study period, which may have influenced levels of posttraumatic stress symptoms. Furthermore, because the PCL-5 referred to participants’ self-identified most distressing event, the source of the reported PTSS cannot be attributed specifically to their interpreting work. Moreover, trauma exposure was operationalized only indirectly through participation in trauma-focused psychotherapy as a binary variable. This measure does not capture important aspects such as the frequency, duration, intensity, or cumulative burden of exposure to traumatic material and may therefore have underestimated variability in work-related trauma exposure. Finally, participant attrition and missing data across measurement time points may have reduced statistical power and introduced bias if participants who discontinued the study differed systematically from those who completed all assessments. Consequently, descriptive differences between measurement occasions may partly reflect changes in the composition of the available sample rather than solely within-person change over time. Finally, specific sample characteristics—such as the low proportion of interpreters with refugee experience—may further limit the transferability of the findings to other interpreter populations.

The small sample size and the limited number of observations reduced the statistical power of the mixed-effects models, particularly for detecting small effects. Therefore, non-significant findings, especially for trauma-informed interpreting competence and involvement in trauma-focused psychotherapy, should not be interpreted as evidence of no association. Accordingly, the absence of statistically significant associations should not be interpreted as evidence that no relationship exists but rather as preliminary findings that require replication in larger, adequately powered samples. The wide confidence intervals for some predictors indicate uncertainty in the estimated effects and suggest that Type II error cannot be ruled out. Future studies with larger samples are needed to replicate these findings and to more precisely estimate the role of competence-related and trauma-exposure-related factors.

### Implications

The present findings tentatively suggest that addressing process-related challenges in psychotherapeutic interpreting may be beneficial for interpreter well-being. Such measures may also have potential relevance for the quality of interpreter-mediated psychotherapy; however, psychotherapy quality and therapeutic outcomes were not assessed in the present study, and this possibility should therefore be regarded as a practical rationale rather than an observed effect. In particular, clear role definitions, structured collaboration between therapists and interpreters, and access to supervision may support lower perceived work-related distress. Practical measures could include structured pre-session briefings to clarify therapeutic goals and interpreter responsibilities, explicit role agreements within the therapeutic triad, and post-session debriefings to reflect on emotionally challenging sessions. Monitoring interpreter stress and providing supervision could identify perceived work-related distress early and facilitate targeted support. Trauma-focused training may further support interpreters, particularly when combined with measures that strengthen role clarity and collaboration within psychotherapeutic settings (Müller et al., 2023).

Importantly, training should not focus solely on interpreters but should also prepare mental healthcare professionals for working effectively in interpreter-mediated settings. Such training may address the respective roles and responsibilities within the therapeutic triad, effective communication and collaboration with interpreters, and the appropriate use of pre-session briefings and post-session debriefings (Bundesweite Arbeitsgemeinschaft der Psychosozialen Zentren für Flüchtlinge und Folteropfer (BAfF), 2022; Tribe and Thompson, 2022). Where therapists are able to participate in selecting an interpreter, the interpreter’s qualification and suitability for the specific therapeutic context should be considered. Relevant factors may include proficiency in the required language or dialect, interpreting competence, understanding of professional roles and confidentiality, and familiarity with mental healthcare contexts. Depending on the individual service user and therapeutic context, preferences concerning characteristics such as gender may also warrant consideration, as may confidentiality concerns in small language communities (Tribe and Thompson, 2022). In trauma-focused psychotherapy, appropriate preparation for working with trauma-related content and awareness of the potential emotional demands associated with interpreting such material may be particularly relevant (Bundesweite Arbeitsgemeinschaft der Psychosozialen Zentren für Flüchtlinge und Folteropfer (BAfF), 2022).

From a research perspective, future studies should aim to develop and validate trauma-specific instruments tailored to interpreters in psychotherapeutic settings and to examine the effectiveness of role clarification, collaboration strategies, training, and supervision using longitudinal or controlled designs. Future research could additionally examine whether training mental healthcare professionals in interpreter-mediated psychotherapy and specific procedures for matching interpreters to service users and therapeutic contexts are associated with improved collaboration within the therapeutic triad and lower perceived work-related distress among interpreters. Such efforts could inform guidelines that address both interpreter well-being and the quality of psychotherapeutic care.

## Data Availability

The datasets presented in this article are not readily available because given the sensitive and potentially identifiable participant information, the datasets generated and analyzed during this study are not publicly available but are available on request from the corresponding author. Requests to access the datasets should be directed to monja.herold@ku.de, https://www.ku.de/bettercare.
