# Supplementary material for: Work-related distress among interpreters working in interpreter-mediated psychotherapy: a 1-year follow-up
Source: Front Psychol. 2026 Oct 14;17:1883184. doi: 10.3389/fpsyg.2026.1883184 (PMC13713788; doi:10.3389/fpsyg.2026.1883184)
Supplement: Supplementary file 1 [file Supplementary_file_1.DOCX]

**Appendix**

[Appendix A: Sensitivity analysis using an ordinal mixed-effects model	2](#_Toc237090168)

[Appendix B1: Missingness and data structure for the multiple-imputation analysis	3](#_Toc237090169)

[Appendix B2: Hierarchical linear mixed-effects model predicting perceived work-related distress following multiple imputation (N = 68 participants, 182 observations).	4](#_Toc237090170)

# Appendix A: Sensitivity analysis using an ordinal mixed-effects model

| Predictor | B | SE | z | p | 95% CI (low) | 95% CI (high) |
| --- | --- | --- | --- | --- | --- | --- |
| MTP | -0.45 | 0.26 | -1.77 | .076 | -0.95 | 0.05 |
| PTSS | 0.42 | 0.19 | 2.22 | .026 | 0.05 | 0.80 |
| Difficulties process | 0.74 | 0.21 | 3.52 | < .001 | 0.33 | 1.15 |
| Difficulties content | 0.17 | 0.20 | 0.871 | .387 | -0.22 | 0.56 |
| Competence | 0.12 | 0.20 | 0.62 | .533 | -0.26 | 0.51 |
| Trauma-focused  therapy | 0.64 | 0.69 | 0.93 | .353 | -0.71 | 1.99 |

Note. *B* = unstandardized regression coefficient (log odds); *SE* = standard error; *CI* = 95% confidence interval. Perceived work-related distress was the dependent variable. MTP = measurement time point; PTSS = posttraumatic stress symptoms. Likert-type predictor variables were z-standardized before analysis; therefore, the coefficients represent the expected change in the log odds of reporting a higher level of perceived work-related distress associated with a one-standard-deviation increase in the respective predictor. The binary predictor (*Trauma-focused psychotherapy*) was entered on its original scale. Threshold coefficients are omitted for clarity. *p* < .05, *p* < .01, *p* < .001.

# Appendix B1: Missingness and data structure for the multiple-imputation analysis

| Occasion | T0 | T2 | T3 | Total |
| --- | --- | --- | --- | --- |
| Scheduled person-occasion assessments | 68 | 68 | 68 | 204 |
| Entire assessment occasion not completed | 3 | 5 | 14 | 22 |
| Unique person-occasion records present | 65 | 63 | 54 | 182 |
| Perceived work-related distress missing | 14 | 26 | 22 | 62 |
| Process-related difficulties missing | 14 | 26 | 22 | 62 |
| Content-related difficulties missing | 14 | 26 | 22 | 62 |
| Observed questionnaire data | 51 | 37 | 32 | 120 |

**Note.** Missingness in perceived work-related distress, process-related difficulties, and content-related difficulties occurred jointly because these variables were derived from the same questionnaire; there were no cases of partial missingness across these three variables. PTSS, trauma-informed interpreting competence, involvement in trauma-focused psychotherapy, and measurement occasion were complete within the 182 existing person-occasion records. The 22 entirely missed assessment occasions were not generated for the multiple-imputation procedure.

| Predictor | B | SE | df | t | p | 95% CI (low) | 95% CI (high) |
| --- | --- | --- | --- | --- | --- | --- | --- |
| (Intercept) | 2.36 | 0.28 | 65.99 | 8.46 | < .001 | 1.81 | 2.92 |
| MTP | -0.19 | 0.13 | 43.83 | -1.47 | .149 | -0.46 | 0.07 |
| PTSS | 0.18 | 0.10 | 68.61 | 1.81 | .075 | -0.02 | 0.38 |
| Difficulties process | 0.35 | 0.11 | 63.51 | 3.35 | .001 | 0.14 | 0.56 |
| Difficulties content | 0.10 | 0.10 | 78.01 | 1.08 | .283 | -0.09 | 0.29 |
| Competence | 0.05 | 0.11 | 48.30 | 0.46 | .647 | -0.17 | 0.27 |
| Trauma-focused  therapy | 0.24 | 0.23 | 40.66 | 1.06 | .297 | -0.22 | 0.71 |

# Appendix B2: Hierarchical linear mixed-effects model predicting perceived work-related distress following multiple imputation (N = 68 participants, 182 observations).

**Note.** *B* = unstandardized regression coefficient; *SE* = standard error; *CI* = 95% confidence interval. Perceived work-related distress was the dependent variable. PTSS = posttraumatic stress symptoms. Predictors measured on Likert-type scales were z-standardized before analysis; therefore, coefficients represent the expected change in perceived work-related distress associated with a one-standard-deviation increase in the respective predictor. The binary predictor (*Involvement in trauma-focused psychotherapy*) was entered on its original scale.
